# The Story of the Insane from Year to Year

**Published:** 1895-02-09

**Authors:** 


### THE STORY OF THE INSANE FROM YEAR TO YEAR.

(7TH SERIES.)

### DONEGAL DISTRICT ASYLUM.

The accommodation is given at 422 beds, and 408 patients were in residence at the beginning of the year, which number had increased to 431 on December 31st. There were 129 admissions, 38 recoveries, 13 discharged relieved, and 48 deaths. The recoveries stand at 29·4 per cent. of the admissions, and the deaths at 11·4 per cent. of the average number resident. Domestic trouble caused the insanity in 4 of the year's admissions, intemperance in drink in 8, and hereditary influence in 32, while 57 are put down as of unknown origin. An "educational table" is given on page 33, and we learn from it that of the patients in the asylum at the end of the year 114 could neither read nor write, 66 could read only, and only 9 are returned as well educated. The usual water supply failed totally, and typhoid fever broke out. There were 32 cases, and these, coupled with the absence of a proper supply of water and a crowded asylum, must have given Dr. Moore many an anxious moment. The average cost per head per annum was £21 6s. 7d.

### NOTTINGHAM COUNTY ASYLUM.

Only 307 patients were in residence at the beginning of the year, and at the close of the year the number had risen to 334. There were 115 admissions, 40 recoveries, and 39 deaths. The recoveries were 34·78 per cent. of the admissions, and the deaths 11·74 per cent. of the average number resident. Domestic trouble caused the insanity in 7 of the admissions, intemperance in drink in 24, and hereditary influence in 38. Cerebro-spinal diseases caused 14 of the deaths, phthisis 6, and small-pox 1. Small-pox, which had viit ed the asylum 11 years previously, made its appearance again, and 10 people were seized by it, but only 1 died. Since the outbreak a detached hospital for infectious diseases has been built in the grounds, but during the epidemic Dr. Aplin must have had his hands full of work and his mind laden with anxiety. The visitors "beg to state that Dr. Aplin and those who are working under his superintendence do all they can to promote the welfare and recovery of their patients." The weekly charge to the unions is 9s. 11d. per head.

### ROYAL ASYLUM, PERTH.

The number of patients in this asylum is just under 100. During the year 1893 there were 34 admissions, 19 recoveries, 10 discharged relieved, and 6 deaths. The percentage of recoveries calculated on the admissions is 55·90—a very high percentage indeed; while the deaths, calculated on the average number resident, stands at 6·31, which is considerably below the average. Regarding the causes of the insanity in the admissions of the year, we find that insane or neurotic heredity accounts for 24, intemperance in drink for 4, and domestic trouble for 5. As to the alleged increase of insanity in our time, Dr. Urquhart does not seem to believe that there is any alarming increase, nor does he expressly record his belief that there is an increase at all. He thinks it is a complex question, and the advantages of civilisation may be counter-balanced by the wear and tear of modern life. Exhaustion of nervous energy may follow; but Dr. Urquhart well observes, "It is not the work of the world that lays this heavy burden on mankind so much as worries and cares; it is not the limitations of the social contract so much as the sudden and turbulent liberation of forces which marks off the nineteenth century from the ages that have gone before." Here, as in so many other asylums, nowadays the attendants and nurses have been trained and educated to take a higher interest in the patients. Three obtained the certificate of the Medico-Psychological Association, making a total of 8 of the staff who have successfully gone through the examinations. The weekly cost per patient is 37s. The ordinary minimum rate of board is £60 per annum, but it is most satisfactory to find that 38 patients were maintained at lower rates, and that £425 were in this way charitably expended during the year. The Commissioners say "the management, both medical and general, is in the hands of a skilful physician, devoted to his work."

